# Resveratrol Inhibits Cisplatin-Induced Epithelial-to-Mesenchymal Transition in Ovarian Cancer Cell Lines

**DOI:** 10.1371/journal.pone.0086987

**Published:** 2014-01-22

**Authors:** Sébastien Baribeau, Parvesh Chaudhry, Sophie Parent, Éric Asselin

**Affiliations:** Research Group in Molecular Oncology and Endocrinology, Department of Medical Biology, Université du Québec à Trois-Rivières, Trois-Rivières, Québec, Canada; Kinghorn Cancer Centre, Garvan Institute of Medical Research, Australia

## Abstract

**Background:**

Many patients diagnosed with ovarian cancer experience recurrence and metastasis, two aspects that will often cause their demise. Epithelial-to-mesenchymal transition (EMT) is a key process involved in cancer progression. With increasing evidence linking Cisplatin and EMT, we wanted to identify a compound able to counter EMT progression when cancer cells are treated with Cisplatin.

**Methodology/Principal Findings:**

Cell death was evaluated by cytometry with Annexin V/PI staining in A2780 and A2780CP cells. Ovarian cancer cell lines were treated with Cisplatin (24 h, 10 µM) and different concentrations of Resveratrol to evaluate its effect on Cisplatin-induced EMT using Western Blot and RT-PCR analysis. Morphological studies and wound healing assay to evaluate cell motility were performed using 72 h Cisplatin treatment with A2780 and A2780CP cells. Densitometry was done on Western Blot and PCR results, and statistical significance was determined using One-Way ANOVA followed by Tukey post-hoc test. Our results show that Cisplatin induced EMT-associated morphological changes in the A2780 ovarian cancer cell line and to a lesser extent in its Cisplatin-resistant counterpart A2780CP. Resveratrol caused cell death in A2780 and A2780CP cell lines in an apoptotic-independent manner. Resveratrol inhibited Cisplatin-induced Snail expression by reducing the Erk pathway activation, reverted morphological changes induced by Cisplatin and decreased cell migration.

**Conclusions:**

These results indicate that Resveratrol has interesting potential to prevent Cisplatin-induced EMT in ovarian cancer cells. By increasing cell death, it also represents an inviting approach as adjuvant therapy to be used with chemotherapy. Using Erk pathway inhibitors could also prove helpful in ovarian cancer treatment to reduce the risk of metastasis.

## Introduction

Ovarian cancer is the seventh most common cancer and the third most common amongst gynaecological cancers in canadian women. Ovarian cancer is also the gynaecological cancer with the highest mortality rate and a 5-year survival rate estimated to only 15–25% [1]. This can be explained by the fact that patients affected by ovarian cancer often already have a high-stage disease at the moment of diagnosis [2], [3]. The usual treatment for ovarian cancer consists of surgical cytoreduction followed by platinum-based chemotherapy [4]. Despite initial response to the treatment, many patients will relapse and eventually be affected by metastases and ultimately meet their demise.

Epithelial-to-mesenchymal transition (EMT) is a physiological process that occurs during embryonic development and occasionally in adults, for example during wound healing [5]. EMT is a phenomenon during which cells will undergo a transition from an epithelial phenotype to a more motile and invasive mesenchymal phenotype, rendering them able to invade tissues and form metastases. The main hallmark of EMT is the loss of E-cadherin, a junction protein typically expressed in epithelial cells. In most cases, E-cadherin loss is mediated by transcriptional repressors, and mutations of the gene or the protein are not common events [6]. The most commonly involved repressors include Snail, Slug, and ZEB1 that directly bind to the E-cadherin promoter to repress its transcription [7], [8]. Many factors are known to induce EMT, including cytokines such as TGF-β [9] or the MAPK-Erk pathway [10]. A recent study on ovarian cancer reported Cisplatin as an inducer of EMT [11].

Snail and Slug are transcription factors mainly known for their involvement in EMT where they repress the expression of epithelial markers, such as E-cadherin and Claudin-1, and increase the expression of mesenchymal markers, such as ZEB1 and MMP-9 [12]–[16]. They can also repress the expression and function of the tumor suppressor p53 and promote chemoresistance [17], [18]. During EMT progression, it is believed that Snail will be the first factor to become active to initiate the transition whereas Slug would be expressed in later stages to allow the cells to retain their mesenchymal characteristics [19]. ZEB1 is another important promoter of EMT by repressing ZO-1 and E-cadherin [20], but can also be involved in increasing the proliferation rate of cells [21].

Resveratrol (trans-3,4′,5-trihydroxystilbene) is a natural compound produced in many plants including red grapes [22], and subsequently present in wine, known for its antioxidant and its protective effects on the cardiovascular system and against cancer in which it can inhibit multiple stages of the disease [23]. During the last years, these many effects placed Resveratrol in the spotlight of research.

In this study, we investigated the impact of Resveratrol on Cisplatin-induced EMT in ovarian cancer. We found that cells co-treated with Resveratrol and Cisplatin did not show the characteristic features of EMT, as Resveratrol treatment abrogated Cisplatin-induced Snail protein and mRNA expressions in a dose-dependent manner. This involved the Erk pathway, which was inhibited by Resveratrol and its involvement was confirmed via specific MEK1/2 inhibition with U0126. Resveratrol also blocked morphological changes in A2780 and A2780CP cells and decreased the migration ability of A2780 and A2780CP cells during a wound healing assay. Similar inhibition of migration was observed in OVCAR-3 and SKOV-3 cells. We also propose β-catenin as a marker of resistance to Cisplatin-induced apoptosis as it was cleaved only in Cisplatin-sensitive cell lines.

## Methods

### Reagents

Dulbecco's modified Eagle medium-F12 (DMEM-F12), and Bovine growth serum (BGS) were purchased from HyClone (South Logan, Utah). Gentamycin, Cisplatin, and Resveratrol were purchased from Sigma-Aldrich (St. Louis, MO). The MEK1/2 inhibitor and the Senescence β-galactosidase staining kit were purchased from Cell Signaling Technology (Danvers, MA). Dead cell apoptosis kit with annexin-V/PI, Trizol reagent and Moloney murine leukemia virus (M-MLV) reverse transcriptase were purchased from Invitrogen (Carlsbad, CA).

### Cell culture and treatment

Human ovarian cancer cell lines A2780 and A2780CP (resistant to Cisplatin) cells were a kind gift from Dr G. Peter Raaphorst (Ottawa Regional Cancer Center, Ottawa, Canada) and were originally described in [24]. OVCAR-3 and SKOV-3 cell lines were purchased from the ATCC. A2780 and A2780CP cells were cultured in DMEM-F12 medium supplemented with 2% BGS. OVCAR-3 cells were cultured in RPMI-1640 medium and SKOV-3 cells were cultured in McCoy's medium both supplemented with 10% FBS. Gentamycin (50 µg/mL) was added to all culture media.

To study the impact of Resveratrol on Cisplatin-induced EMT, A2780 and A2780CP cells were co-treated with Resveratrol and 10 µM Cisplatin for 24 h. To study the impact of the Erk pathway on Cisplatin-induced EMT, ovarian cancer cell lines were pretreated with 20 µM U0126 for 1 h, then Cisplatin was added to the culture medium for 24 h at a final concentration of 10 µM. DMSO was used as control for U0126 and Resveratrol.

### Detection of cell death by flow cytometry

A2780 and A2780CP cells were treated with Resveratrol and Cisplatin for 24 h. At harvest, cells were centrifuged at 500 rpm for 5 minutes, washed with PBS and then centrifuged 300 rpm for 5 minutes. Cell death was evaluated with Annexin-V-FITC and propidium iodide (PI) double staining using dead cell apoptosis kit according to the manufacturer's protocol. Fluorescence was read using a Cytomics FC 500 MPL flow cytometer (Beckman Coulter). Positive cells for Annexin-V and/or PI were considered as dead cells.

### MTT assay

Proliferation of A2780 and A2780CP cells was evaluated by MTT assay. Cells were plated in duplicate in 96-well plates and left to adhere overnight. The following day, cells in growth phase were treated with 0–60 µM Resveratrol with or without 10 µM Cisplatin for 24 h in 100 µL culture medium. 4 h before the end of treatment, 10 µL of MTT solution was added to each well and plates were placed in the incubator. 4 h later, 100 µL solubilisation solution (10% SDS in 0.01 M HCl) was added to each well and plates were left in the incubator overnight. The following day, the absorbance was read at A_595nm_ with a Fluostar Optima plate reader.

### Western Blot

Cells were harvested after 24 h Resveratrol-Cisplatin or U0126-Cisplatin treatment and washed twice with phosphate-buffered saline (PBS). Cells were then lysed in RIPA lysis buffer containing complete protease inhibitor cocktail (Roche Applied Science) and subjected to three freeze/thaw cycles at -20°C. Cells were then centrifuged and the supernatant was collected. Protein concentration was estimated using the BioRad DC Protein assay. Equal amount of proteins (20–40 µg) were then separated onto SDS-PAGE and transferred to nitrocellulose membranes (BioRad, Hercules, CA). Membranes were blocked in 5% non-fat skimmed milk in PBS-Tween 20 0.05% (PBS-T) for 1 h before incubation with primary antibody (diluted 1∶1000 in PBS-T/milk) overnight at 4°C (all antibodies are from Cell Signaling Technology, Danvers, MA, except GAPDH from Abcam (Cambridge, MA)). Primary antibody targeting Snail was incubated 2 h at room temperature and HRP-conjugated-GAPDH was incubated 45 minutes at room temperature. After incubation, the membranes were washed four times in PBS-T before incubation with horseradish peroxidase conjugated secondary antibody (BioRad, Hercules, CA) diluted 1∶3000 in PBS-T/milk. Membranes were washed four times in PBS-T and the antibodies were revealed using SuperSignal West Femto substrate (Thermo Scientific, Rockford, IL), as described by the manufacturer using UVP Bio Imaging System. GAPDH was used as loading control.

### Cell morphology

Cells were grown in 6-well plates and then treated with 2.5 µM Cisplatin and/or 60 µM Resveratrol for 72 h in A2780 and A2780CP cells to allow enough time for EMT morphological changes to occur. After treatment, cells were washed with fresh medium to remove dead cells. Finally, cells were observed under a Carl Zeiss Axio observerZ1 microscope to determine their morphology and picture were taken in phase contrast microscopy using 20X magnification. Cells presenting mesenchymal characteristics, with a more fibroblast-like morphology and cellular protusions, were considered as having undergone EMT.

### Wound healing assay

To evaluate cell motility, cells were plated in 24-well plates and grown to confluency. A sterile tip was used to create a scratch in the cell layer. Cells were then treated with 2.5 µM Cisplatin and 60 µM Resveratrol and pictures were taken at appropriate times to evaluate wound closure. Wounds were evaluated using ImageJ software to measure the wound area at different time points. The percentage of wound closure was calculated as wound area at a given time compared to the initial wound surface. Pictures shown are representative of three independent experiments performed in duplicates.

### Reverse Transcriptase PCR (RT-PCR)

Total RNA was extracted from treated cells using Trizol Reagent according to the manufacturer's instructions. 500 ng of RNA was reverse transcribed using M-MLV reverse transcriptase and oligo(dT) primers. The reverse-transcribed RNA was then amplified by PCR using specific primers. GAPDH was used as an internal control. Human Snail was amplified using the sense primer 5′-TCGGAAGCCTAACTACAGCGA-3′ and antisense primer 5′-AGATGAGCATTGGCAGCGAG-3′ (fragment length 140 bp). Human TCF8-ZEB1 was amplified using sense primer 5′-TTACACCTTTGCATACAGAACCC-3′ and antisense primer 5′-TTTACGATTACACCCAGACTGC-3′ (fragment length 100 bp). Human Vimentin was amplified using sense 5′-CGAAAACACCCTGCAATCTT-3′ and antisense primer 5′- CTGGATTTCCTCTTCGTGGA-3′ (fragment lenght 133 bp). GAPDH was amplified using sense 5′-GTCAGTGGTGGACCTGACCT-3′ and antisense primer 5′-GACTTGACAAAGTGGTCG-3′ (fragment length 139 bp). GAPDH was used as control.

### Senescence-associated β-galactosidase staining

Cells were plated in 6-well plates and left to adhere overnight. The following day, cells were treated with 60 µM Resveratrol and 2.5 µM Cisplatin for 72 h. After the treatment, cells were washed to remove non-adherent cells and stained with the Senescence β-galactosidase staining kit according to the manufacturer's instructions. Cells were then counted under a light microscope to evaluate the percentage of senescent cells which appeared as blue cells.

### Densitometry and Statistical analysis

Densitometric analysis was done on the Western Blot results and PCR pictures using Quantity One software (BioRad) to determine the abundance of proteins or amplified mRNA studied. In both cases, GAPDH was used as control. In wound healing assays, wound area was mesured using ImageJ software. Wound closure was evaluated by comparing the wound area at a given time to the initial wound area. Comparison between treatments was performed using GraphPad PRISM software version 5.00 (San Diego, CA) using one-way ANOVA analysis and the *post hoc* Tukey's test. Statistical significance was accepted when p<0.05. All experiments performed in this study were repeated three independent times.

## Results

### Resveratrol induces cell death in ovarian cancer cells

**Figure 1:**
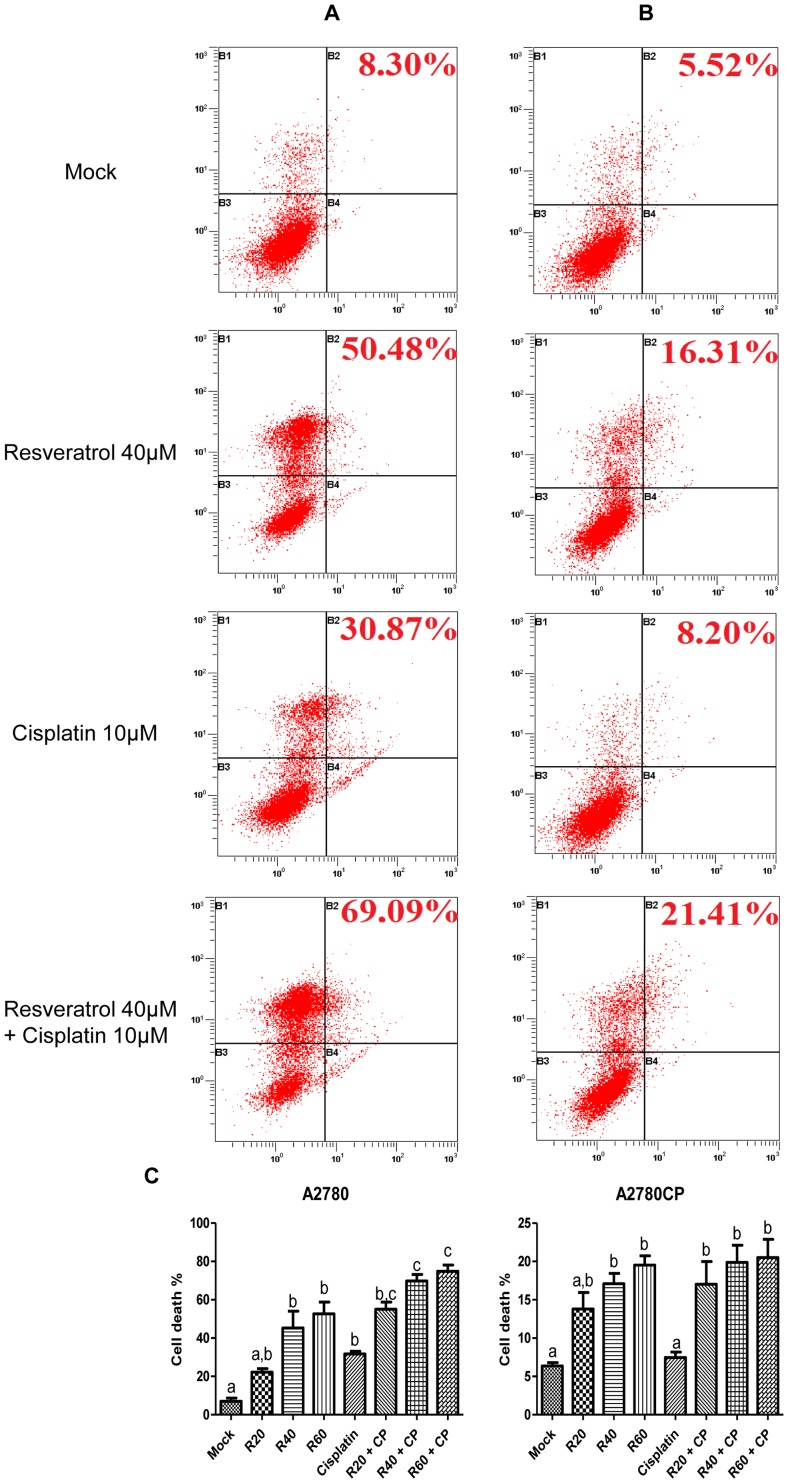
Resveratrol induces cell death and potentiates Cisplatin cytotoxicity. Cell death levels were evaluated by flow cytometry with Annexin-V and PI staining in A2780 and A2780CP cells. Representative flow cytometry results are shown for A2780 (A) and A2780CP cells (B) treated with 10 µM Cisplatin and 40 µM Resveratrol. In the upper right corner, percentage of cell death is shown, corresponding to apoptotic and necrotic cell death. Annexin-V and PI negative cells (B3) are non-apoptotic. Annexin-V positive and PI negative cells (B4) are in the early stages of apoptosis. Annexin-V positive and PI positive cells (B2) are in late apoptosis. Annexin-V negative and PI positive cells (B1) are necrotic cells. Cell death percentage is shown in (C) for both cell lines treated with increasing doses of Resveratrol up to 60 µM with or without Cisplatin 10 µM. Results shown are representative of three independent experiments. In graphs, columns identified with different letters are significantly different (p<0.05). In graphs, mock: untreated cells, R20: Resveratrol 20 µM, R40: Resveratrol 40 µM, R60: Resveratrol 60 µM, CP: Cisplatin, R20CP: Resv. 20 µM + Cisplatin, R40CP: Resv. 40 µM + Cisplatin, R60CP: Resv. 60 µM + Cisplatin.

Treatment of A2780 and A2780CP ovarian cancer cells with Resveratrol increased cell death in a dose-dependent manner as shown by the proportion of PI-positive cells. The low proportion of Annexin-V staining in Resveratrol-treated samples but not in Cisplatin-treated samples suggests that Resveratrol might induce cell death through a different mechanism or occur faster than Cisplatin-induced cell death (Figure 1).

**Figure 2:**
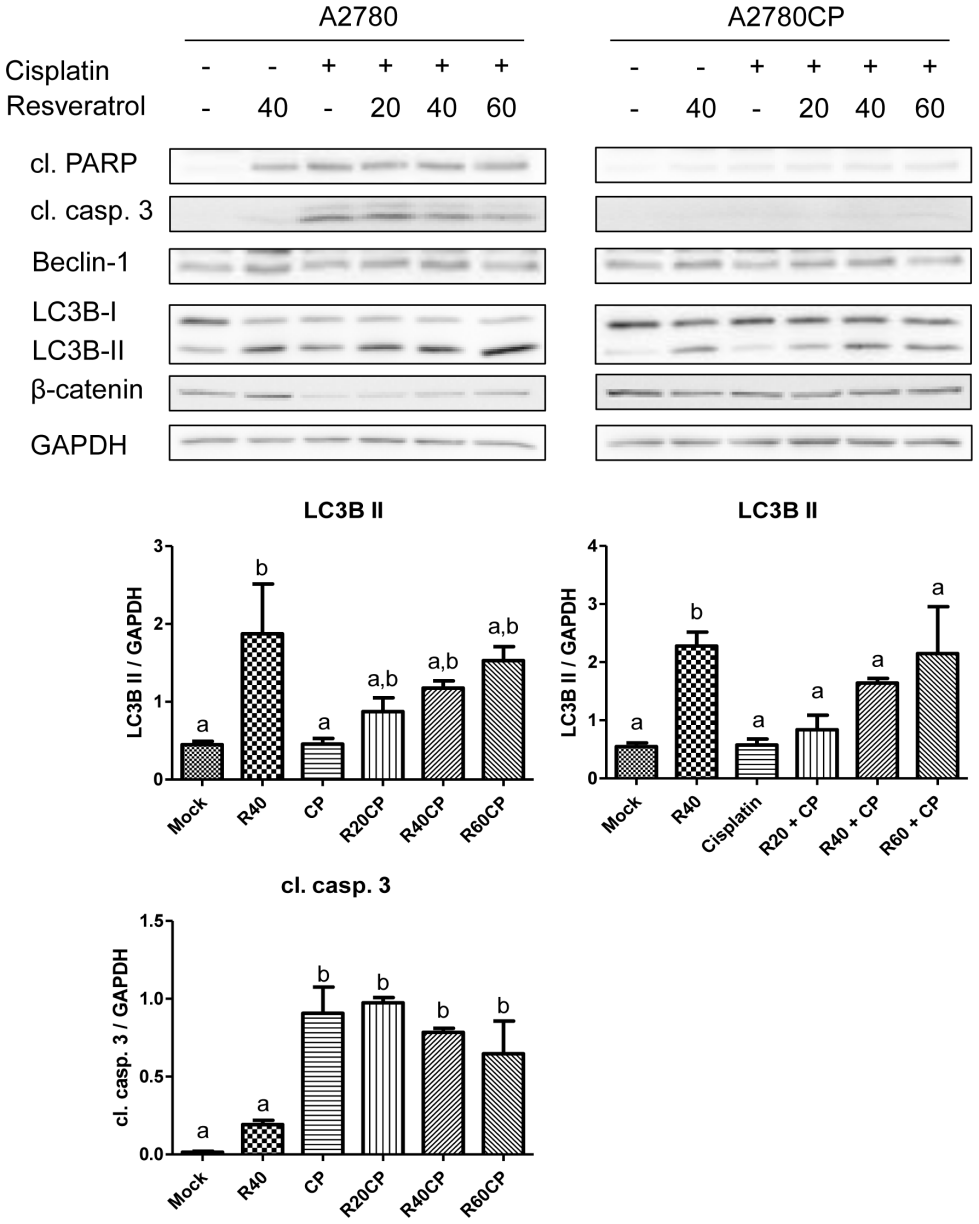
Resveratrol potentiates Cisplatin-induced cell death through autophagy. The mechanism of cell death was studied in A2780 and A2780CP cells in response to Cisplatin and Resveratrol. The absence of increase in cleaved PARP levels and caspase-3 cleavage when higher doses of Resveratrol are used suggests Resveratrol would induce cell death through a mechanism independent of apoptosis. The significant increase in LC3BII suggests Resveratrol could induce cell death through autophagy. Densitometry analysis is shown under Western Blot for each cell line. Results shown are representative of three independent experiments. In graphs, columns identified with different letters are significantly different (p<0.05). In graphs, mock: untreated cells, R40: Resveratrol 40 µM, CP: Cisplatin, R20CP: Resv. 20 µM + Cisplatin, R40CP: Resv. 40 µM + Cisplatin, R60CP: Resv. 60 µM + Cisplatin.

We investigated apoptosis and autophagy as potential cell death mechanisms. In A2780 cells, Cisplatin induced an increase in caspase-3 and PARP cleavage, indicating induction of cell death through apoptosis. By contrast, treatment of A2780 or A2780CP cells with Resveratrol did not affect the levels of cleaved caspase-3 despite the presence of dead cells, strengthening the possibility that a mechanism other than apoptosis is involved (Figure 2). We then decided to study autophagy induction by evaluating the expression levels of LC3B-II and Beclin-1, two proteins known for their involvement in autophagy progression. Resveratrol caused an increase in LC3B-II levels in both cell lines, supporting results from Opipari *et al*. showing autophagy as a potential mechanism responsible for Resveratrol-induced cell death [25]. In our model, we did not observe an increase in Beclin-1 levels, which is already expressed at relatively high levels in A2780 and A2780CP cell lines.

### Resveratrol potentiates Cisplatin-induced decrease in cell proliferation

**Figure 3:**
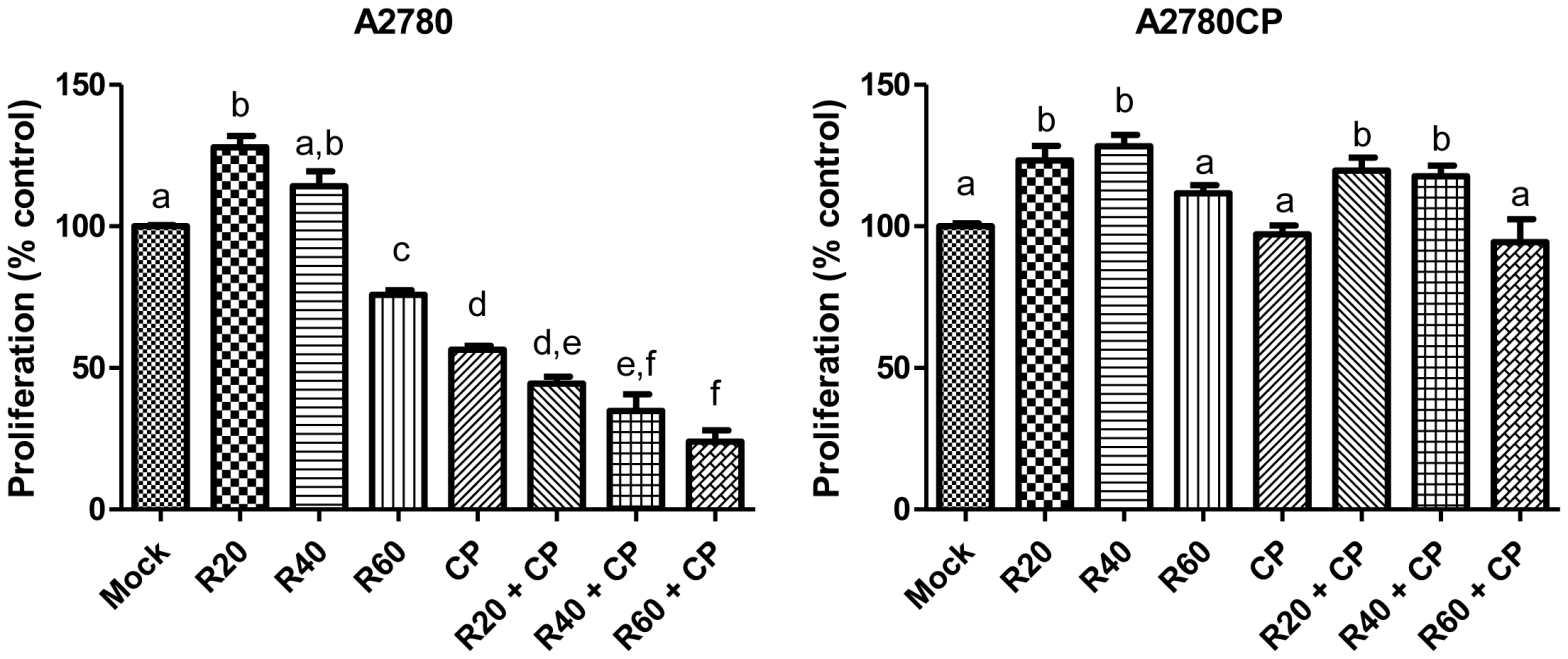
Resveratrol decreases proliferation of Cisplatin-sensitive A2780 cells but not Cisplatin-resistant A2780CP cells. MTT assay was performed to evaluate A2780 and A2780 cell lines proliferation in response to 10 µM Cisplatin and increasing doses up to 60 µM of Resveratrol. A2780 cells proliferation was increased by Resveratrol at 20 µM and decreased at 60 µM while all doses suggest an increase in proliferation in A2780CP cells. Resveratrol also potentiates Cisplatin decrease of proliferation in A2780 cells. Graph bars with different letters are statistically different (p<0.05). In graphs, mock: untreated cells, R20: Resveratrol 20 µM, R40: Resveratrol 40 µM, R60: Resveratrol 60 µM, CP: Cisplatin, R20CP: Resv. 20 µM + Cisplatin, R40CP: Resv. 40 µM + Cisplatin, R60CP: Resv. 60 µM + Cisplatin.

We next sought to evaluate the effect of Resveratrol on the proliferation of A2780 and A2780CP cell lines. As shown in Figure 3, low doses of Resveratrol (20–40 µM) slightly increases proliferation of both cell lines despite the cell death associated to this treatment. At the highest dose tested (60 µM) Resveratrol induced a slight decrease in proliferation in A2780 cells and almost no change in A2780CP cells. In Cisplatin-sensitive A2780 cells, Resveratrol potentiates the decrease in cell proliferation observed when Cisplatin is used as treatment suggesting a synergistic action between these two compounds. This was not observed in Cisplatin-resistant A2780CP cells but the highest Resveratrol dose did not increase proliferation when used in combination with Cisplatin.

### Resveratrol inhibits Cisplatin-mediated EMT induction in ovarian cancer cell lines

**Figure 4:**
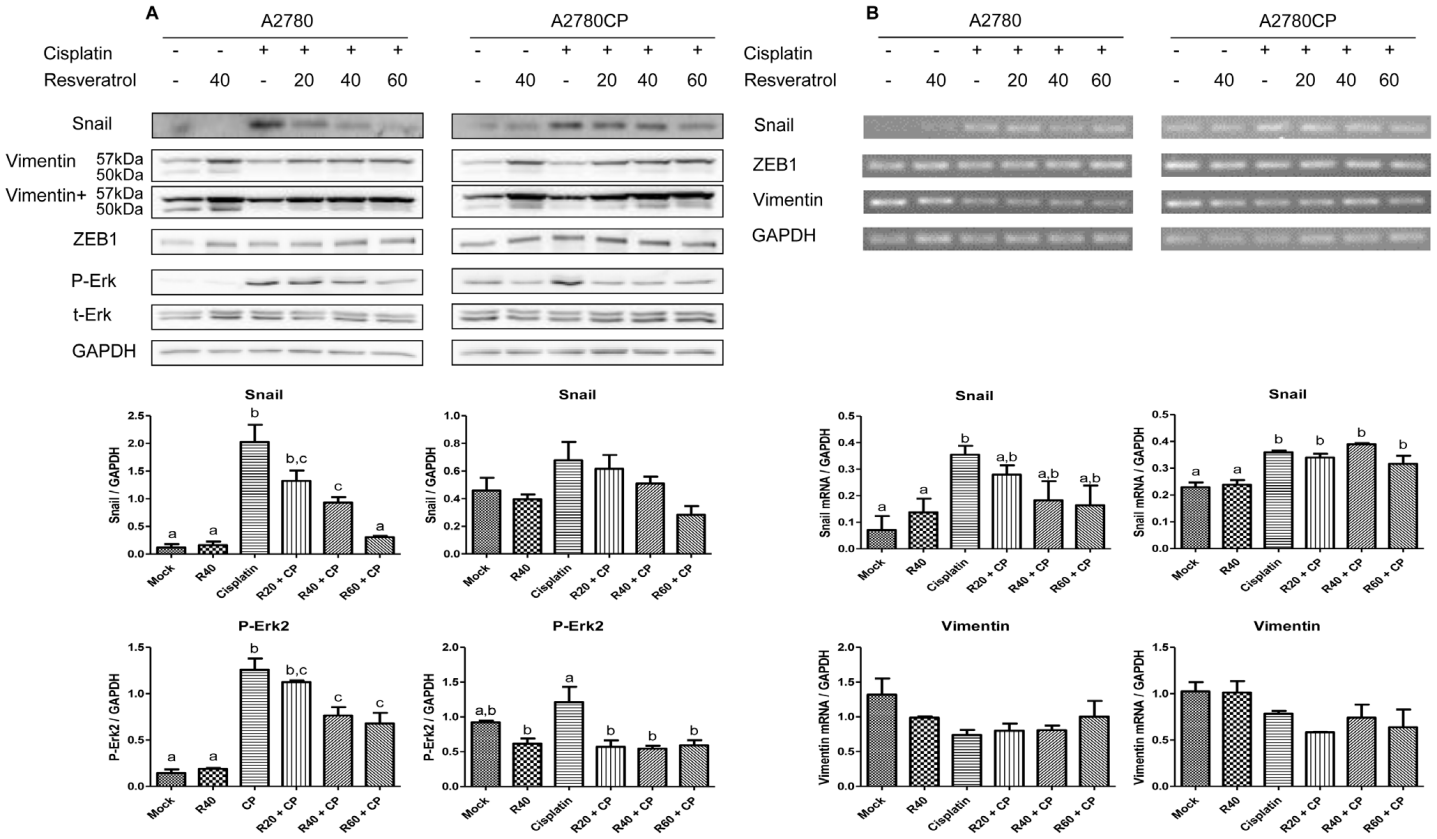
Resveratrol reduces Cisplatin-induced Snail expression. Western Blot analysis of EMT markers Snail, Vimentin and ZEB1 in A2780 and A2780CP (A) cells in response to 10 µm Cisplatin with or without increasing doses of Resveratrol. Densitometric analysis is shown under each cell line Western Blots. Resveratrol inhibits Cisplatin-induced Snail expression in a dose-dependent manner but slightly increases ZEB1 and Vimentin levels. The Resveratrol dose-dependent decrease in P-Erk2 levels suggests the involvement of the Erk pathway in Resveratrol-induced Snail downregulation. The EMT markers Snail, Vimentin and ZEB1 expression were analysed by RT-PCR in A2780 and A2780CP (B) cells treated with 10 µm Cisplatin with or without increasing doses of Resveratrol. Resveratrol or Cisplatin treatment had no effect on ZEB1 and Vimentin mRNA levels. Vimentin and ZEB1 densitometry is shown under each cell line. Snail mRNA expression was decreased only in A2780 cells, suggesting another mechanism could be involved in A2780CP cells. Images shown are representative of three independent experiments. In graphs, columns identified with different letters are significantly different (p<0.05). In graphs, mock: untreated cells, R40: Resveratrol 40 µM, CP: Cisplatin, R20CP: Resv. 20 µM + Cisplatin, R40CP: Resv. 40 µM + Cisplatin, R60CP: Resv. 60 µM + Cisplatin.

To assess the impact of Resveratrol on Cisplatin-induced EMT, A2780 and A2780CP cells were co-treated with Resveratrol and Cisplatin. As expected, treating the cells with Cisplatin increased the protein levels of Snail, Vimentin and ZEB1, three of the main markers of EMT. Treating the cells with Cisplatin and Resveratrol decreased the expression levels of Snail, but did not show this effect on Vimentin and ZEB1 expression. By contrast, these two proteins were slightly increased when cells were exposed to Resveratrol alone (Figure 4A). The decrease in Snail levels in A2780 treated with Resveratrol and Cisplatin correlates with a decrease in Snail mRNA levels.However, this was not the case in A2780CP cells where no significant change was observed in Snail mRNA levels between samples treated with Cisplatin and those treated with both Cisplatin and Resveratrol, suggesting some post-translational mechanisms might also be involved in Snail downregulation in these cells (Figure 4B). ZEB1 and Vimentin mRNA levels remained mostly unaltered in cells treated with either Resveratrol, Cisplatin or both agents. In our cell model, we could not assess EMT progression based on downregulation of E-cadherin because A2780 and A2780CP cells do not express this protein.

### Activation of the MAPK Erk is associated with Cisplatin-induced EMT

**Figure 5:**
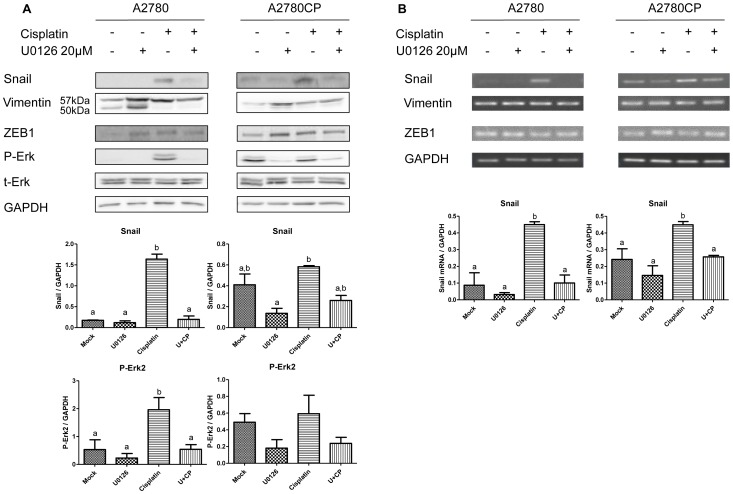
Erk inhibition abrogates Cisplatin-induced EMT in ovarian cancer cell lines. A2780 and A2780CP cell lines were pre-treated with U0126 to inhibit Erk activity and treated with Cisplatin for 24 h. Western Blot are shown in (A). Corresponding densitometry is shown under each cell line. Erk inhibition reduced Cisplatin-induced Snail expression but slightly increased ZEB1 and Vimentin expression in both cell lines. Corresponding PCR results are shown in (B). Snail mRNA decrease in U0126+Cisplatin treated samples suggests Snail regulation by Cisplatin involves gene transcription. Results shown are representative of three independent experiments. In graphs, columns identified with different letters are significantly different (p<0.05). In graphs, mock: untreated cells, CP: Cisplatine, U+CP: U0126+ Cisplatin.

We next investigated the mechanism by which Resveratrol counters Cisplatin-induced Snail expression. The MAPK/Erk pathway is one of the possible mechanisms that can be involved in EMT induction in cells. In A2780 and A2780CP cells, Erk2 phosphorylation was increased in response to Cisplatin. The activation of this pathway was significantly inhibited by Resveratrol, corresponding to a decrease in Snail levels in A2780 and A2780CP cell lines (Figure 4). To further confirm the involvement of Erk in the increase of Snail levels in response to Cisplatin, we used U0126, a MEK1/2 specific inhibitor (Figure 5). Pretreatment of cells with this inhibitor significantly reduced the activation levels of Erk2 in A2780 and A2780CP cells, which also correlated with a significant decrease in Snail protein in both cell lines. Slight increases in the protein levels of Vimentin and ZEB1 were also observed (Figure 5A). Snail mRNA levels were decreased when both cells lines were treated with Cisplatin and U0126, suggesting Cisplatin-induced Snail expression through the Erk pathway requires transcription of the Snail gene. ZEB1 and Vimentin mRNA levels did not show significant changes when A2780 and A2780CP cells were treated with Cisplatin and U0126 alone or in combination (Figure 5B). These results support Resveratrol-induced EMT inhibition through the Erk pathway.

### Resveratrol blocks Cisplatin-induced morphological changes

**Figure 6:**
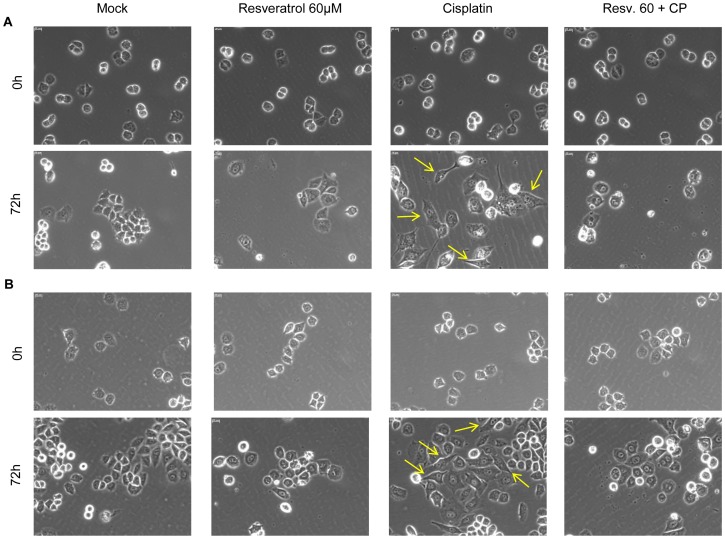
Resveratrol blocks EMT morphological changes induced by Cisplatin. A2780 (A) and A2780CP (B) cell lines were treated for 72 h with 2.5 µM Cisplatin with or without 60 µm Resveratrol to allow EMT morphological changes to become apparent. Cisplatin induced cellular morphological changes reminiscent of EMT in A2780 cells evident after 72 h, which were abrogated when Resveratrol was added to the treatment. Resveratrol exerted the same effect on A2780CP cells although the morphological changes induced by Cisplatin were less pronounced. Cells were observed under 20X magnification, scale bar in the upper left corner of the pictures represent 20 µm. Arrows indicate examples of cells having undergone an EMT. Images shown are representative of three independent experiments.

In order to avoid excessive Cisplatin-induced cytotoxicity in A2780 cells, we decided to use 2.5 µM Cisplatin to assess morphological changes in A2780 and A2780CP cells for a 72 h treatment. Treatment of ovarian cancer cells with Cisplatin induced morphological changes reminiscent of EMT as the cells lost their epithelial cobblestone-like morphology to acquire a more elongated fibroblast-like shape. Cellular protusions also appeared in Cisplatin-treated cells compared to control samples. Both A2780 and A2780CP cells showed these modifications indicating their progression through EMT. To test the effect of Resveratrol on this phenomenon, we used 60 µM, the concentration at which Snail downregulation was the most evident. Addition of Resveratrol to Cisplatin treatment blocked these morphological changes as the cells did not acquire any of the characteristic mesenchymal morphological features (Figure 6). It is also interesting to note that Resveratrol-treated cells became bigger and displayed an irregular appearance suggesting another cellular transformation, such as a senescence program, could be occurring [26].

### Resveratrol induces senescence

**Figure 7:**
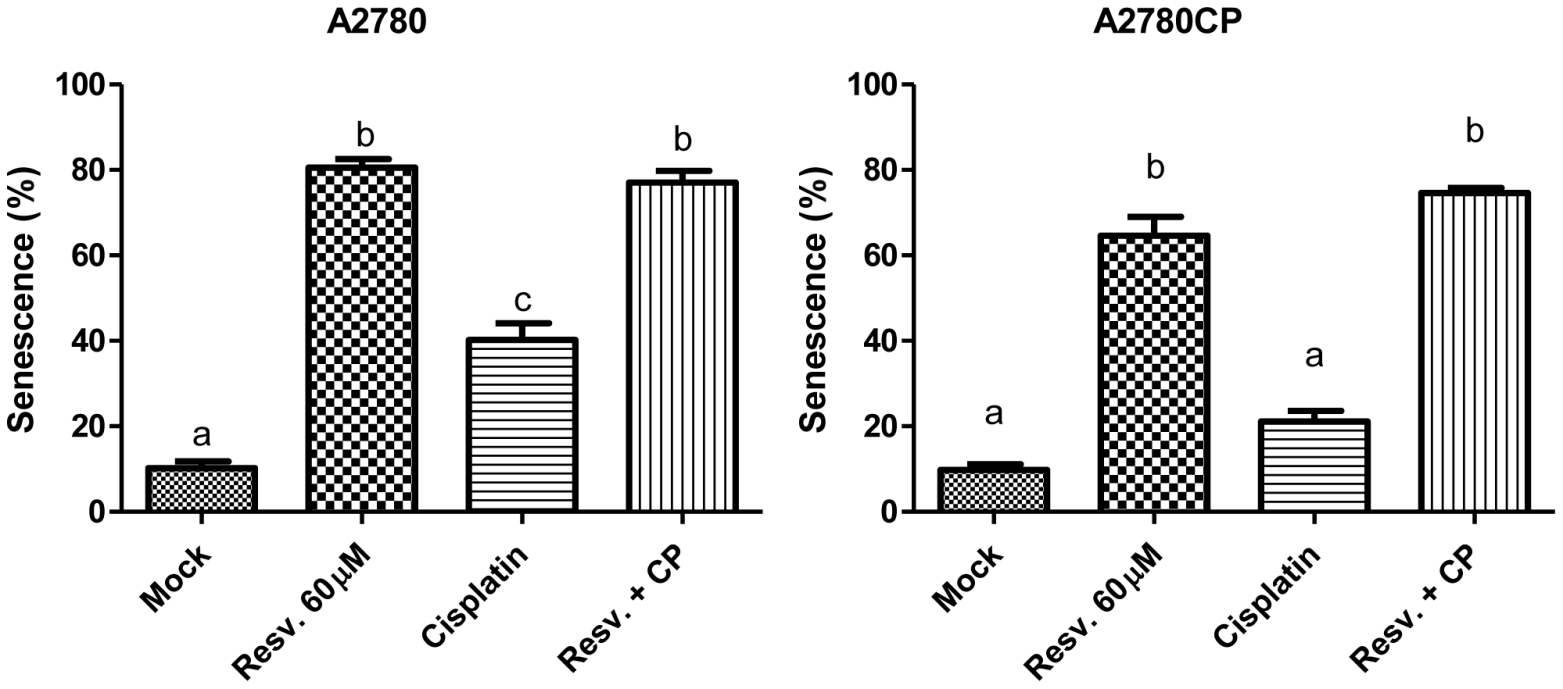
Resveratrol and Cisplatin induce senescence in ovarian cancer cell lines. Treatment of A2780 and A2780CP cells with Resveratrol significantly increased the proportion of senescent cells. Cisplatin induced a significant increase of senescence in A2780 cells. Cells were stained for β-galactosidase activity and counted under a light microscope to assess the percentage of senescent (blue) cells. In graphs, columns identified with different letters are significantly different (p<0.05). The analysis was performed with three independent experiments.

We evaluated senescence induction in our cell lines after Cisplatin and Resveratrol treatment and observed a significant increase in Resveratrol-induced senescence in both cell lines after 72 h, with a more pronounced effect in A2780 cells (Figure 7). We also observed higher senescence levels in A2780 cells treated with Cisplatin and suggest that continuous exposure to Cisplatin might induce an oxidative stress leading to senescence in these Cisplatin-sensitive cells. Senescence induction in A2780 and A2780CP cells could be another mechanism involved in the inhibition of cisplatin-induced EMT by Resveratrol.

### Resveratrol inhibits cell migration

**Figure 8:**
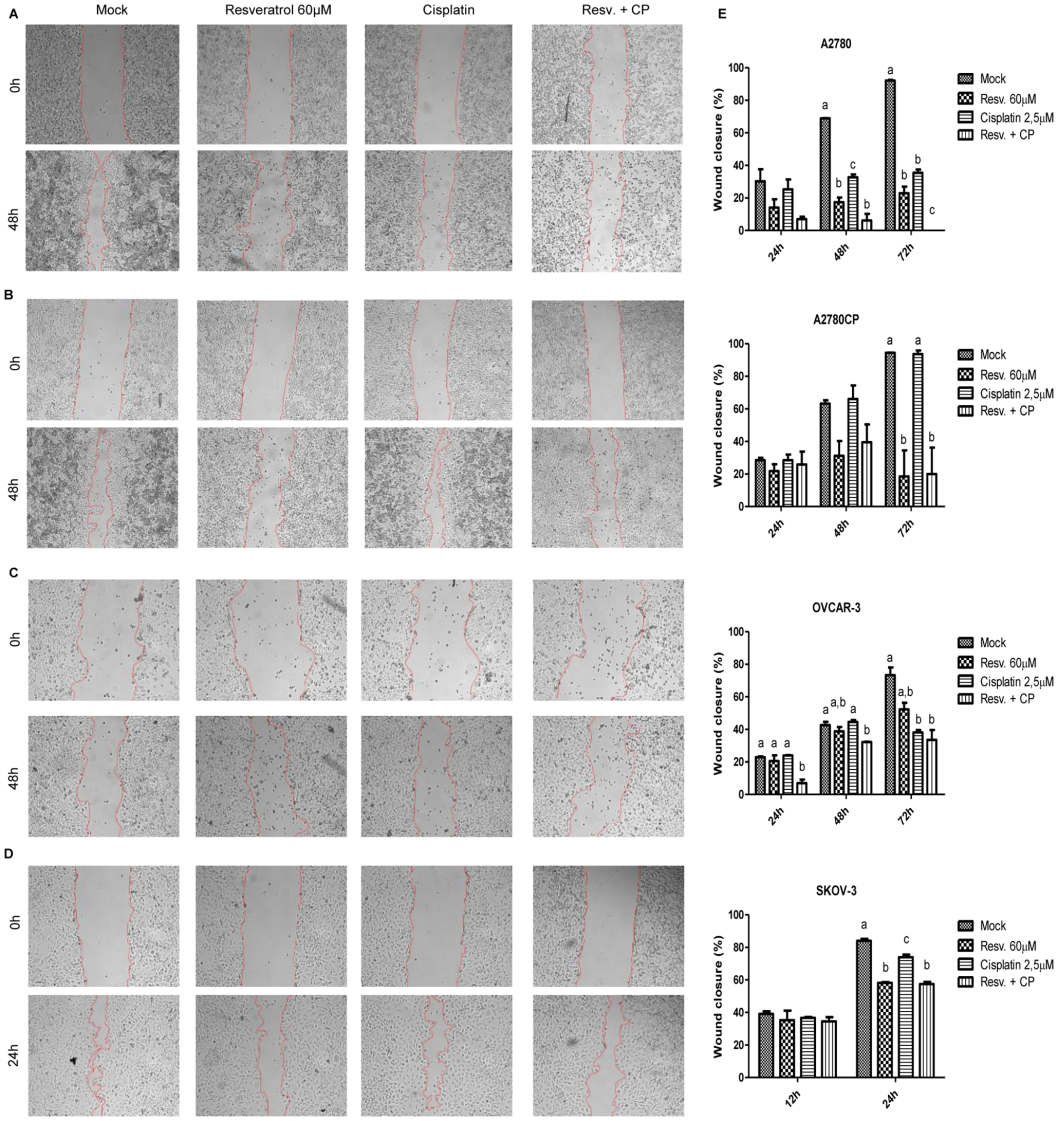
Wound healing capacity of ovarian cancer cell lines is impaired by Resveratrol. The migratory ability of A2780 (A), A2780CP (B), OVCAR-3 (C), and SKOV-3 (D) cells was evaluated by wound healing assay. A scratch was made with a sterile tip in a confluent layer of cells and wound closure was observed after 24, 48, and 72 h of Cisplatin and Resveratrol treatment for A2780, A2780CP, and OVCAR-3 cells, and 12 and 24 h for SKOV-3, corresponding to the moment where the wound was almost completely closed. In both cell lines, Resveratrol decreased the migration of the cells in the wound, preventing its closing after 72 h. Representative pictures are shown at t = 48 h. Graphical representation of wound closure after the different time points studied is shown in (E). Results shown are representative of three independent experiments performed in duplicates. In graphs, treatments are compared within each time point studied and columns identified with different letters are significantly different (p<0.05).

Considering the morphological transformation occurring in A2780 and A2780CP cells, we next evaluated the migration ability of these cells with a wound healing assay. Cells were treated with 2.5 µM Cisplatin, to avoid excessive cytotoxicity in A2780 cells, and 60 µM Resveratrol. In both cell lines, 72 h were required for the wound to completely close up in untreated cells, suggesting that these cell lines don't have a very mobile phenotype. Resveratrol treatment clearly inhibited cell migration at every time point where wound closure was evaluated, reducing the wound closure to only 20–25% after 72 h for both cell lines whereas the control cells had almost completely closed the wounds (Figure 8A and 8B). Resveratrol's action would not be affected by Cisplatin in A2780CP cells where this agent exerted the same effect on the cells with or without Cisplatin.

Furthermore, we investigated the potential of Resveratrol to inhibit the migration ability of OVCAR-3 cells, an epithelial cell line expressing E-cadherin and without basal expression of ZEB1 or Vimentin which shows little migration potential, and SKOV-3 cells, a mesenchymal cell line showing basal expression of Vimentin and ZEB1 as well as a mesenchymal morphology and high migration potential despite basal E-cadherin expression (Figure S1). Similar to A2780 and A2780CP cells, OVCAR-3 cells required at least 72 h to close the wound. In this cell line, Resveratrol significantly reduced the closure of the wound after 72 h and at every time point studied when used in combination with Cisplatin. In SKOV-3 cells, only 24 h were required to observe 80–85% wound closure and Resveratrol significantly decreased the migration of these cells when used alone or in combination with Cisplatin at this time point, indicating that this phytoalexin possesses the capacity to reduce the migration of cells that possess a high basal migration potential as well as cells with low migration potential (Figure 8C and 8D). Results observed in OVCAR-3 and SKOV-3 cells correlate with those observed in A2780 and A2780CP respectively, where sensitivity to cisplatin is also similar.

To address the possibility of an increase in the invasion potential of the cells, we performed a zymography assay in A2780. A2780CP, OVCAR-3 and SKOV-3 cells with Cisplatin and Resveratrol treatments. In all four cell lines, we only detected pro-MMPs at different levels, highest pro-MMP activity was observed in OVCAR-3 cells and lowest activity was observed in A2780 and A2780CP cells, which displayed almost no pro-MMP activity. We did not observe any active form of MMPs in this assay for any of the cell lines studied. Furthermore, Resveratrol and Cisplatin treatment, alone or in combination, did not affect pro-MMPs activity and did not induce active MMPs, suggesting that these treatment do not alter the invasion capacity of the cells (data not shown).

## Discussion

Recently, Haslehurst *et al*. demonstrated an involvement for Snail and Slug, two of the main EMT activating transcription factors, in the chemoresistance of ovarian cancer [27]. Moreover, the demonstration that Cisplatin induces EMT [11] brings about a new challenge: how to fight back? Indeed, preventing EMT induction by Cisplatin shows an interesting potential to help patients overcome chemotherapy resistance and metastatic dissemination. In order to try to answer to this problem, we evaluated the impact of Resveratrol, a natural compound known for its wide array of beneficial effects [28], on Cisplatin-induced EMT in ovarian cancer.

First, we assessed cell death in response to Cisplatin and Resveratrol in A2780 and A2780CP cells. In A2780 cells, Resveratrol increased cell death when used as a single treatment and when used in combination with Cisplatin where it potentiated Cisplatin-induced cell death. By contrast, in Cisplatin-resistant A2780CP cells, Resveratrol induced cell death to a lesser extent compared to A2780 cells when used as a single treatment and failed to sensitise A2780CP cells to Cisplatin-induced death in our conditions where cell death remained the same as Resveratrol treatment alone. Our results suggest apoptosis is not responsible for Resveratrol-induced cell death as there is no increase in cleaved caspase-3. An alternative mechanism of cell death could be autophagy, which was previously shown to be involved in ovarian cancer cell death by Resveratrol [25]. We also show an increase in LC3B-II in both cell lines, suggesting cell death could be caused by this cellular process despite no increase in Beclin-1 levels, which could be due to the time point selected.

We suggest that β-catenin could be used as a marker to evaluate cancer sensitivity to Cisplatin. In our model, only the sensitive cell line (A2780) displayed β-catenin downregulation when treated with Cisplatin (Figure 2), consequent with a previous report demonstrating β-catenin cleavage by caspase-3 during apoptosis [29]. However, β-catenin could not be used as a marker for caspase-independent cell death mechanisms as its downregulation requires caspase-3 activation.

Despite an increase in cell death, Resveratrol increased cellular proliferation when used at low doses (20 and 40 µM) in our cell lines. We previously demonstrated that resveratrol can bind to the estrogen receptor α (ERα) in uterine cancer cells [30]. However, A2780 and A2780CP cell lines are ERα negative but we hypothesize that Resveratrol might bind to the estrogen receptor β (ERβ). This could also explain the synergistic action between Cisplatin and Resveratrol in A2780 cells since binding of Resveratrol to ERβ could increase gene transcription and then expose DNA to Cisplatin and increase its efficiency to induce cell death.

We confirmed results published earlier in a study on ovarian cancer reporting that Cisplatin increases EMT markers in ovarian cancer [11]. Cisplatin caused an increase in the protein levels of Snail, ZEB1 and Vimentin in both cell lines studied as soon as 24 h of treatment. Such a quick increase in Snail levels suggests that Cisplatin induces an EMT program even after a one-time/short-term treatment while TGF-β, a cytokine known for its ability to induce EMT, usually requires 72 h to achieve this process [31]. The increase in Snail levels was reverted by Resveratrol in a dose-dependent manner, which would involve the Erk pathway, as confirmed by MEK1/2 specific inhibition by U0126. However, Resveratrol and U0126 still increased Vimentin and ZEB1 levels.

Despite their lack of E-cadherin expression, A2780 and A2780CP cells represent an interesting model to study the induction of EMT since they possess many of the characteristics of epithelial cells such as an epithelial morphology that is altered by Cisplatin, thus allowing cells to gain a fibroblast-like mesenchymal morphology (Figure 6), and a low migration capacity in basal conditions (Figure 8). By contrast, SKOV-3 cells, which express basal E-cadherin levels, do not display an epithelial phenotype as they display fibroblast-like morphology (Figure S1) and possess an evident capacity to close a wound under basal conditions (Figure 8). Thus, we believe that A2780 and A2780CP cells, despite their defaults in their expression of some of the EMT markers, are an interesting model to study EMT induction by cisplatin as they display many epithelial characteristics. Other research groups also used A2780 cells as an epithelial model in their studies. Recently, Fangfang Du *et al.* demonstrated the potential involvement of EMT in the acquisition of paclitaxel resistance in A2780 cells [32].

Furthermore, Cisplatin-resistant A2780CP cells, which are less sensitive to Resveratrol-induced cell death, support that results observed in A2780 cells regarding EMT inhibition by Resveratrol are not due to excessive cell death and that Resveratrol possesses the potential to inhibit EMT in this cell model.

Another clue that Resveratrol inhibits Cisplatin-induced EMT came from the cellular morphology. Indeed, A2780 and A2780CP cells treated with Cisplatin alone displayed an evident morphology change from an epithelial cobblestone-like morphology to a more elongated morphology with cellular protusions reminiscent of cells having undergone EMT. However, cells treated with both Cisplatin and Resveratrol did not show such morphological modifications.

Recent studies in lung and colon cancer showed that Resveratrol induces senescence when used at doses in a range corresponding to the doses used in our study when treated for at least 10–12 days [33], [34]. We were then interested in studying senescence induction by Resveratrol in A2780 and A2780CP cells. According to our results, Resveratrol induces a senescent phenotype in A2780 and A2780CP cells after 72 h of treatment in an interesting proportion of the cells. This phenomenon could also be a mechanism through which Resveratrol inhibits Cisplatin-induced EMT in A2780 and A2780CP cells since induction of senescence would inhibit the initiation of other cellular programs such as EMT and then block the effects of Cisplatin on these two ovarian cancer cell lines.

In wound healing assay, Resveratrol treatment displayed a marked decrease in cell migration in A2780 and A2780CP cells. This suggests that Resveratrol is able to inhibit migration, one of the main features acquired by cells undergoing EMT, and could then reduce metastatic potential of ovarian cancers. The decrease in migration observed for OVCAR-3 and SKOV-3 cells also holds potential for a wider application as Resveratrol also reduces the migration ability of another epithelial cell line, as well as a mesenchymal cell lines displaying a stronger migration capacity.

**Figure 9:**
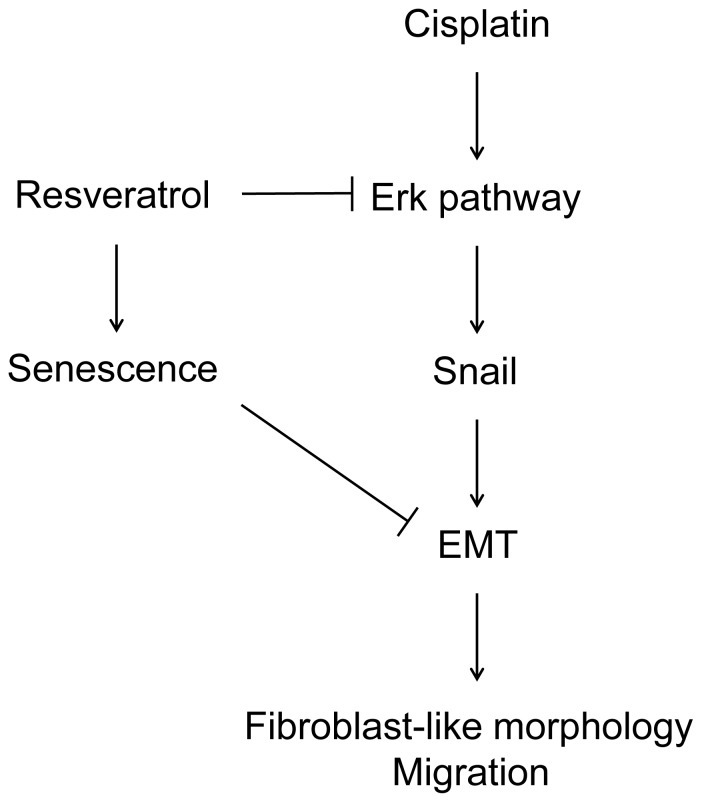
Involvement of Resveratrol in the inhibition of Cisplatin-induced EMT. Treatment of ovarian cancer cell lines with Cisplatin results in the activation of the Erk pathway and subsequent induction of Snail expression and induction of EMT. By contrast, Resveratrol prevents Cisplatin-mediated EMT induction by antagonizing the activation of the Erk pathway, thus preventing Snail levels increase and EMT induction. Furthermore, Resveratrol could also prevent Cisplatin-induced EMT through induction of a senescence program that would block the progression of other cellular differentiation programs like EMT.

Recent studies using other cancer cell lines also support the role of Resveratrol in EMT inhibition. Vergara *et al.* demonstrated that Resveratrol inhibits EMT induction by EGF in MCF-7 breast cancer cell line via inhibition of the EGF-mediated Erk pathway activation [35]. A study from another group also supports the role of Resveratrol in inhibiting EMT induction in cancer cells in A549 lung cancer cells. In their study, the authors report that Resveratrol blocks TGF-β1 induced EMT and reduces the levels of EMT transcription factors Snail and Slug [36]. In our study, we suggest two mechanisms that can be responsible for EMT inhibition by Resveratrol (Figure 9). Resveratrol treatment reduced Cisplatin-mediated activation of the Erk pathway in A2780 and A2780CP cells, thus inhibiting Snail expression. Furthermore, Resveratrol significantly induced senescence in both cell lines, which could act as a dominant protection program in the cell to prevent the activation of other cellular differentiation pathways. In this regard, as a bonus to preventing EMT induction by Cisplatin, Resveratrol could alter cellular proliferation pathways and be a major determinant to prevent cancer progression.The fact that more than 90% of ovarian cancers are of epithelial origin [37] allows EMT to play a determinant role in ovarian cancer progression. Another important aspect of EMT is its possible involvement in the ability of cancer cells to resist to chemotherapeutic agents as well as acquire other important advantages such as resistance to anoikis and evasion from the immune system [38]. Combining these important consequences of EMT, it is clear that this phenomenon is a major factor involved in the high mortality rates associated with ovarian cancer. Considering this, finding new approaches to block EMT induction in cancer shows great promise to improve the outcome for patients. Our results suggest that Resveratrol might be an interesting compound to prevent cancer development or as an adjuvant molecule for cancer treatment once diagnosed.

However, bioavailability of Resveratrol might be a problem since it is quickly metabolised and it might be difficult to reach high local concentrations matching the doses used during *in vitro* studies [39]. To assess this problem it would be important to find alternate routes of administration, such as localised treatment or local injections, to improve Resveratrol's effect on cancer cells. In regard of our results, targeting the Erk pathway also shows interesting promise to decrease cancer progression towards metastasis and could reveal an important asset in the fight against cancer.

## Supporting Information

Figure S1 **Epithelial and mesenchymal markers expressed in OVCAR-3 and SKOV-3 cells and their basal morphology.** Expression of the epithelial marker (E-cadherin) and expression of the mesenchymal markers (ZEB1, Vimentin and Snail) was determined in OVCAR-3 and SKOV-3 cells treated or not with Cisplatin and Resveratrol (A). Basal morphology is shown for both cell lines under their corresponding protein expression profile (B). Despite its E-cadherin expression, SKOV-3 displays morphological characteristics of mesenchymal cells.
(TIF)
